# An Asian viewpoint on the use of vitamin D and calcium in osteoporosis treatment: Physician and patient attitudes and beliefs

**DOI:** 10.1186/1471-2474-11-248

**Published:** 2010-10-26

**Authors:** Siew Pheng Chan, Boyd B Scott, Shuvayu S Sen

**Affiliations:** 1 Department of Medicine, Faculty of Medicine, University of Malaya, Kuala 50603, Lumpur, Malaysia; 2 Merck & Co., Inc., Whitehouse Station, NJ, USA

## Abstract

**Background:**

Osteoporosis treatment guidelines recommend calcium and vitamin D supplementation for both prevention as well as treatment, however, compliance with these guidelines is often unsatisfactory. This study investigated the opinion of Asian physicians and Asian patients regarding vitamin D and calcium and patients' use of both.

**Methods:**

Physicians selected from Malaysia, Taiwan, Philippines, Korea and Singapore were asked to grade the significance of vitamin D and calcium in the treatment of osteoporosis and their patients' use of these supplements. In addition, physicians recruited seven eligible osteoporotic women to answer a questionnaire to determine their use of vitamin D and calcium, and their attitudes and beliefs regarding these supplements.

**Results:**

In total, 237 physicians and 1463 osteoporosis patients completed the questionnaire. The results revealed that 22% of physicians in Malaysia, 12% in Taiwan, 72% in the Philippines, 50% in Korea and 24% in Singapore rated the importance of vitamin D supplementation as being extremely important. For calcium, 27% of physicians in Malaysia, 30% in Taiwan, 80% in the Philippines, 50% in Korea and 38% in Singapore rated the importance as being extremely important. Forty-three percent of patients in Malaysia, 38% in Taiwan, 73% in the Philippines, 35% in Korea and 39% in Singapore rated the importance of vitamin D as being extremely important. For calcium, 69% of patients in Malaysia, 58% in Taiwan, 90% in the Philippines, 70% in Korea and 55% in Singapore rated the importance as being extremely important. In addition, results of the patient questionnaire revealed that only a very small number regularly took both supplements. In addition, the results indicated that, with the exception of patients from the Philippines, the majority of patients had no or infrequent discussion with their physician about vitamin D and calcium.

**Conclusions:**

There is generally suboptimal appreciation by both physicians and patients of the importance of vitamin D and calcium for maintenance of bone health as reflected in the low number of patients who reported regularly taking these supplements. Recognition of this problem should translate to appropriate action to improve education for both physicians and patients, with a goal to increase use of these supplements among Asian patients with osteoporosis.

## Background

The effect of osteoporosis on public health and the resultant financial burden are considerable [1,2]. Until recently the majority of epidemiological studies on osteoporosis have been performed in Western Caucasian populations in which the incidence of osteoporosis is high [2]; but as Asian populations become more aged, similar phenomena are being observed with the incidence of osteoporosis increasing [3-5]. Hip fracture is a significant public health problem today in Asia as in Europe and North America. It has been projected that by the year 2050, 50% of hip fractures worldwide will occur in Asia [6-8]. Although the incidence of hip fracture is generally lower in Asia [9], there is variation in the rate based on ethnicity and geographic location [5,10]. The underlying mortality rate and functional outcomes are very similar to those observed in Western Caucasian populations [10-13]. Compounding the problem of increased incidence of osteoporosis in Asian populations is the findings from epidemiological studies that have identified a high incidence of vitamin D inadequacy[14,15]. Vitamin D deficiency has also been identified in postmenopausal women in Eastern Asia [16].

Effective therapies are now available for the treatment of osteoporosis, most notably the oral bisphosphonates, currently the most commonly used therapeutic modality for this disease [17]. In addition to the various active anti-osteoporosis agents, there is clear evidence that adequate vitamin D and calcium intake also play significant roles in ameliorating the severity of osteoporosis and are necessary for optimal response to pharmacologic intervention [18,19]. Vitamin D and calcium can be obtained directly via the diet or by supplementation [20,21]. A chronically low intake of calcium in the diet decreases bone mass and leads to an increased risk of osteoporosis and bone fracture [22]. Calcium supplementation alone has been demonstrated to increase bone mineral density and to decrease the rate of fracture in postmenopausal women [23-27]. Likewise, addition of supplemental vitamin D to the diet has also been shown to reduce fracture rate in postmenopausal women [28,29]. Combination of both calcium and vitamin D supplementation has been shown to reduce the rate of bone mineral density loss and fracture rates [30-33]. Consequently, most clinical guidelines recommend supplementation with calcium and vitamin D to optimize the efficacy of pharmacologic therapies for osteoporosis [20,34].

Guidelines regarding the recommended daily calcium intake vary according to gender, life stage, and also from region to region [35,36]. However, the present recommended minimum daily calcium intake for adults ranges from 1000-1300 mg [35-37]. Even though calcium is relatively common [35,38] in the diet, supplementation is frequently necessary. Intake is often insufficient due to religious or dietary restrictions, lactose intolerance, etc. It is recommended that if dietary intake of calcium-rich foods is inadequate, supplements containing elemental calcium (calcium citrate and calcium carbonate) should be taken [35]. It should be noted that commonly used multivitamin supplements often do not contain sufficient calcium to make up for deficiency in the diet [35].

Increasing calcium intake alone is insufficient for adequate maintenance of bone mass. Sufficient vitamin D is required for optimal intestinal calcium absorption [39]. Calcium absorption decreases as 25-hydroxyvitamin D (25(OH)D) levels decrease, leading to bone loss [40]. As a result, persistently low levels of vitamin D are linked to reduced bone density and an increase in the risk of fragility fracture [41]. At present, the US National Institutes of Health Institute of Medicine recommends a daily intake of 400 IU of cholecalciferol (vitamin D_3_) for women ages 51-70 years, increasing to 600 IU for women over age 70 [36]. The Scientific Committee for Food of the Commission of the European Community presently recommends 400 IU daily for those 65 years of age or older [42]. Despite the recommendations of these bodies, many women being treated for osteoporosis do not regularly take sufficient vitamin D supplementation and have low serum 25(OH)D levels, the clinical parameter used to measure vitamin D status [29,43]. Unlike calcium, which is readily available in a number of dietary sources, dietary sources of vitamin D are more restricted (oily fish, some fortified foods, and egg yolks). Apart from these limited dietary sources of vitamin D, the alternative source is exposure to sunlight. However, exposure to sunlight is often limited for adequate synthesis due to lack of sunlight in Northerly latitudes or due to the avoidance of exposure for cosmetic/cultural reasons and concerns about the risk of skin cancer. As a result, most people are likely to require supplementation to be vitamin D replete [44-46]. Although vitamin D is necessary for regulation of calcium metabolism and maintenance of normal bone health, a significant body of evidence indicates that vitamin D deficiency/inadequacy is common [47,48]. Notably, low levels of vitamin D are frequently observed in older females, the population most affected by osteoporosis [49-51]. Despite the clear evidence for vitamin D inadequacy playing an important role in aggravating osteoporosis, this involvement is not widely understood, recognized or appreciated by physicians as well as patients [39,45,52,53].

A recent study, Incidence and Characterization of inadequate clinical Responders in Osteoporosis (ICARO), observed that lack of compliance with treatment and insufficient supplementation of calcium and vitamin D have a major impact on response to therapy in osteoporosis [54]. It has been reported in a recently conducted questionnaire that while patients are knowledgeable about the role of calcium as a bone-building agent, but they are less knowledgeable about the role of vitamin D in this process [55]. This study and others have highlighted the lack of compliance among patients who were aware of the importance of both vitamin D and calcium [34]. Although a number of European studies have described differences in attitudes and beliefs between physicians and their patients on matters regarding osteoporosis, current therapies, and compliance [56,57], few have explored these issues in Asian populations. The goal of this study was to measure physicians' and patients' attitudes and beliefs regarding vitamin D and calcium and their importance in the treatment of osteoporosis and to detail perception and actual compliance with guidelines regarding these supplements in Asian populations.

## Methods

### Questionnaire design

Throughout 2006, a total of 237 randomly selected physicians in Malaysia (n = 37), Taiwan (n = 50), the Philippines (n = 50), Korea (n = 50) and Singapore (n = 50) were requested to enroll seven consecutive eligible osteoporosis patients who were willing to take part in a telephone questionnaire concerning the significance of vitamin D and calcium to osteoporosis therapy. As it can be complex to enroll physicians to answer a questionnaire of this nature, a sample of 50 physicians per country was considered to be a reasonable minimum number to acquire results with a good degree of statistical robustness. The physicians were requested to enroll the first seven osteoporosis patients who met the inclusion criteria. This was thought to be an acceptable undertaking for the physicians and would provide the opportunity to analyze patient results by sub-groups. This method was used to help randomize the mix of subjects enrolled, hence ensuring that the participating subjects were nationally representative for the enrollment criteria. Statistical power was not provided by the physician or patient sample sizes, and these population sizes were used by the questionnaire designers' previous knowledge of what sample sizes are achievable and reasonable, given the goals and budget of the study. Physicians included such specialties as General Medicine, Internal Medicine, Gynecology, Orthopedic Surgery, Geriatrics, and Rheumatology, specialties that are most likely to treat patients with osteoporosis.

A convenient sample of physicians was chosen from physician licensure databases from each country participating in the study. Only post-menopausal women ≥ 50 years of age who had been diagnosed with and were being treated for osteoporosis were eligible to participate. Exclusion criteria for both physicians and patients included participation in any market research involving women's health during the 3-months prior to participating in the questionnaire and/or having a relative who is employed by a pharmaceutical company.

The questionnaire was translated to the local language and validated in the countries that the study was undertaken. The questionnaire was conducted by a skilled local contractor.

This survey was conducted in accordance with the Declaration of Helsinki guidelines for good clinical practice. Patients were informed about the survey by their physician and voluntarily contacted the survey administrators to participate. Patients were instructed about the nature of the survey and gave their consent to participate. Since participation did not affect patient care, Institutional Review Board and ethics committee review was not required. Physician and patient response data were collected, managed, and secured by Research International Healthcare, which has defined procedures to maintain the security and confidentiality of the data.

It was determined that General practitioners should see a minimum of 10 patients with osteoporosis each month, whereas specialists were required to see a minimum of 15 osteoporosis patients each month. However, it should be noted that these enrollment criteria were ascertained by self reporting, and no further validation was performed. To make certain of an adequate level of experience, physicians were required to have been in practice for ≥ 3 years or < 30 years, to guarantee a good level of experience, and to be current with recent treatment practices. Physicians' participation in the questionnaire occurred after complete enrollment of the minimum seven suitable patients and only once those patient questionnaires were finalized in order to minimize the risk that physicians might inadvertently or purposefully influence patient answers. Nonetheless, it is possible that patients may have obtained some knowledge of the subject matter from the physician at the time of their enrollment. Physicians had no knowledge of the precise nature of the patient questionnaires or aims of the study prior to the patient interviews; hence it would have been unlikely that physicians could influence patient responses even if desired. However, the authors concede that they cannot guarantee that previous interactions between patient and physician prior to interviewing of either party did not impact responses. During the course of the study, however, a number of physicians did not enroll the required 7 patients, and consequently the patient number criterion (400 per country) was relaxed so that the study could be concluded. The final number of completed patient questionnaires was as follows: Malaysia (n = 251), Taiwan (n = 218), the Philippines (n = 194), Korea (n = 400) and Singapore (n = 400).

The protocol for the questionnaire involved the use of telephone interviews for both physicians and patients using a 16-question questionnaire. The questionnaire for both patient (Additional file 1) and physician (Additional file 2) involved the use of open-ended response items, closed-end questions, and scale items (scale from 1-10, where 1 = not important at all, and 10 = extremely important).

Some of the major questions that this questionnaire was intended to answer were:

1. Are physicians and patients knowledgeable regarding the importance of vitamin D and calcium in the management of osteoporosis?

2. Are osteoporosis patients compliant with their recommended dosing regimens for supplementary vitamin D and calcium?

3. Are physicians aware of their osteoporosis patients' willingness to follow recommended dosing regimens for supplementary vitamin D and calcium?

4. How much information did patients receive regarding the need for vitamin D and calcium in the treatment of osteoporosis?

To ensure that the data collected was valid, quality checks were performed to ensure that answers complied with the basic rules of the questionnaire such as no multi-response answers for single-response questions. Although data were not checked to ensure that the subjects responded reliably to all questions, there are no grounds to assume that subjects did not respond to the best of their knowledge and ability. The questionnaires were designed to collect demographic data and obtain answers regarding the subjects' attitudes and beliefs in regards to vitamin D and calcium in the treatment of osteoporosis.

### Statistical/data analysis

Statistical analysis was performed utilizing a Microsoft Excel-based analysis tool. A standard two-tailed tests set at *p *< 0.01 (99% level of significance) were employed to test data between countries. We determined whether percentages or mean scores were appropriate. In the case of ordinal scales, statistical analyses were performed on differences between percentages for individual answer options, rather than on differences between means. Differences were often performed between percentages rather than means as certain scales are not linear and hence a mean score is irrelevant. Analyses were only performed between countries.

## Results

### Physician and patient characteristics

Two hundred and thirty seven physicians participated: 37 from Malaysia, and 50 each from Taiwan, the Philippines, Korea and Singapore; demographics are shown in Table-1. 1463 osteoporosis patients (251, 218, 194, 400 and 400 from Malaysia, Taiwan, the Philippines, Korea and Singapore respectively) participated in the questionnaire; demographic data are shown in Table 2.

**Table 1:** Physicians demographics

Demographics-physicians		Malaysia (n = 37)		Taiwan (n = 50)		Philippines (n = 50)		Korea (n = 50)		Singapore (n = 50)	
		
		%	Actual	%	Actual	%	Actual	%	Actual	%	Actual
**Medical speciality**	**GP/IM (Internist)**	59	22	14	7	-	-	30	15	50	25
	**Gynecologist**	11	4	2	1	20	10	12	6	4	2
	**Orthopedist**	16	6	60	30	38	19	54	27	30	15
	**Geriatrician**	8	3	18	9	20	10	-	-	4	2
	**Rheumatologist**	5	2	6	3	10	5	4	2	8	4
	**Endocrinologist**	-	-	-	-	-	-	-	-	4	2
	**Other**	-	-	-	-	12	6	-	-	-	-

**Years in practice following medical school**	**4-10**	16	6	36	18	48	24	38	19	24	12
	**11-15**	22	8	28	14	14	7	26	13	24	12
	**16-20**	5	2	20	10	18	9	16	8	22	11
	**21-25**	27	10	12	6	16	8	14	7	22	11
	**26-30**	30	11	4	2	4	2	6	3	8	4

	**30-45**	38	14	60	30	58	29	54	27	62	31
**Age**	**46-55**	41	15	40	20	22	11	38	19	32	16
	**56-65**	22	8	-	-	20	10	8	4	6	3

**Location**	**Urban**	-	-	88	44	-	-	-	-	-	-
	
	**Rural**	-	-	12	6	-	-	-	-	-	-

**Table 2:** Patient demographics

Demographics-patients		Malaysia (n = 251)		Taiwan (n = 218)		Philippines (n = 194)		Korea (n = 400)		Singapore (n = 400)	
		
		%	Actual	%	Actual	%	Actual	%	Actual	%	Actual
	**In past year**	35	89	28	62	56	109	80	320	35	139
**Length of time since diagnosis**	**Last 5 years**	49	123	47	103	37	71	16	64	50	198
	**6-10 years**	11	28	18	39	5	10	4	14	12	47
	**11-15 years**	2	6	4	9	1	1	1	2	4	14
	**16-20 years**	1	2	1	2	1	1	-	-	-	1
	**21+ years**	1	3	1	3	2	2	-	-	-	1

**Taking vitamins or minerals to help treat osteoporosis**	**Yes**	67	167	88	191	87	168	62	249	86	343
	**No**	33	84	12	27	13	23	38	151	14	57

	**50-54**	24	59	34	74	11	22	10	40	27	106
	**55-59**	22	54	17	37	16	32	14	55	23	91
**Age**	**60-64**	17	42	19	41	21	40	21	84	15	59
	**65-69**	13	33	10	22	15	30	19	76	15	60
	**70-74**	12	30	10	22	12	23	19	77	12	49

	**75+**	13	33	10	22	24	47	17	68	9	35

Although each physician was requested to enroll a minimum of seven patients, the enrollment target was not met in Malaysia, Taiwan and the Philippines. Consequently numbers required to achieve statistically significant differences in attitude and belief between Malaysia, Taiwan and the Philippines and other countries is slightly higher than it would have been if enrollment targets had been met. For example if 50% of patients in Korea (*n *= 400) replied 'yes' to a question, whereas 60% replied 'yes' in Singapore (*n *= 400) and Taiwan (*n *= 218), Korea and Singapore would differ at a level of 99% confidence whereas Korea and Taiwan would not (though they would at a level of at least 95%). The physicians were on average 45.5 years old and had been practicing for a mean of 14.8 years. There were no statistically significant differences between countries with respect to mean physician age or mean practice experience. Most were general practitioners or orthopedists. Mean patient age was 63.4 years, and mean length of time since osteoporosis diagnosis was 2.6 years. There were no statistically significant differences between countries with respect to mean patient age or mean length of time since osteoporosis diagnosis.

### Physician and patient awareness of the importance of vitamin D and calcium in the management of osteoporosis

**Figure 1:**
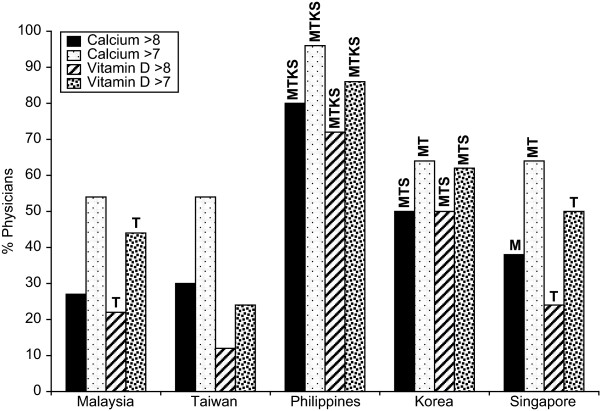
**Importance of specific factors in Osteoporosis management**. M = Malaysia, T = Taiwan, P = Philippines, K = Korea, S = Singapore i.e. In Malaysian data 39TPKS denotes that the number is sig higher than the same response in Taiwan, Philippines, Korea and Singapore.

In response to the question on the importance of vitamin D and calcium in management of osteoporosis, using a scale of 1 to 10 (where 1 = not important at all; and 10 = extremely important), vitamin D was thought to be of high importance (>8) in the management of osteoporosis by 72% of physicians in the Philippines compared with 22% in Malaysia, 12% in Taiwan, 50% in Korea and 24% in Singapore (Figure 1). Calcium was also thought to be of high importance (>8) in the management of osteoporosis by 80% of physicians in the Philippines compared with 27% in Malaysia, 12% in Taiwan, 50% in Korea and 38% in Singapore (Figure 1). Within each country, with the exception of Korea, physicians gave more importance to calcium than to vitamin D.

**Figure 2:**
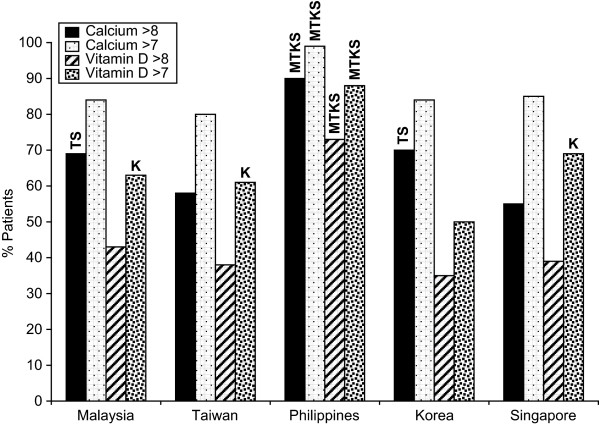
**Importance of specific factors in Osteoporosis management**. M = Malaysia, T = Taiwan, P = Philippines, K = Korea, S = Singapore i.e. In Malaysian data 39TPKS denotes that the number is sig higher than the same response in Taiwan, Philippines, Korea and Singapore.

In response to the same question regarding the importance of vitamin D and calcium in the management of osteoporosis, patients, like physicians, generally considered calcium to be more important than vitamin D (Figure 2). Vitamin D was thought to be of high importance (>8) in the management of osteoporosis by 73% of patients in the Philippines compared with 43% in Malaysia, 38% in Taiwan, 35% in Korea and 39% in Singapore (Figure 2). Calcium was thought to be of high importance (>8) in the management of osteoporosis by 90% of patients in the Philippines compared with 69% in Malaysia, 58% in Taiwan, 70% in Korea and 55% in Singapore

### Compliance with of calcium and vitamin D supplements and physician and patient discussion regarding supplements

#### Malaysia

**Figure 3:**
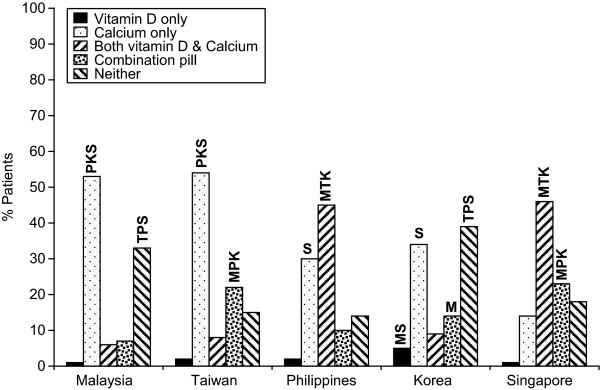
**Supplements taken by patients for osteoporosis**. M = Malaysia, T = Taiwan, P = Philippines, K = Korea, S = Singapore i.e. In Malaysian data 39TPKS denotes that the number is sig higher than the same response in Taiwan, Philippines, Korea and Singapore.

**Figure 4:**
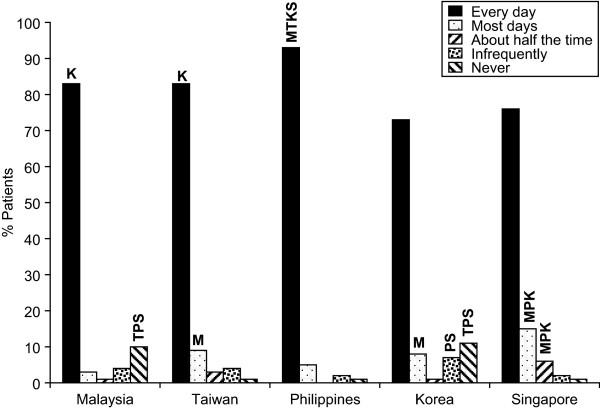
**Frequency at which osteoporosis supplements are taken**. M = Malaysia, T = Taiwan, P = Philippines, K = Korea, S = Singapore i.e. In Malaysian data 39TPKS denotes that the number is sig higher than the same response in Taiwan, Philippines, Korea and Singapore.

**Figure 5:**
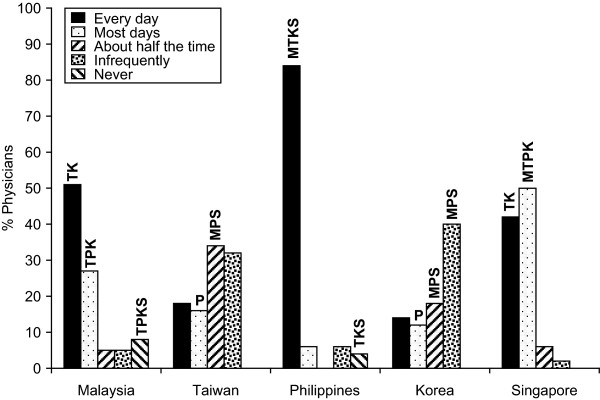
**Frequency at which physicians perceive their patients take vitamin D (or vitamin D+Calcium) supplements**. M = Malaysia, T = Taiwan, P = Philippines, K = Korea, S = Singapore i.e. In Malaysian data 39TPKS denotes that the number is sig higher than the same response in Taiwan, Philippines, Korea and Singapore.

**Figure 6:**
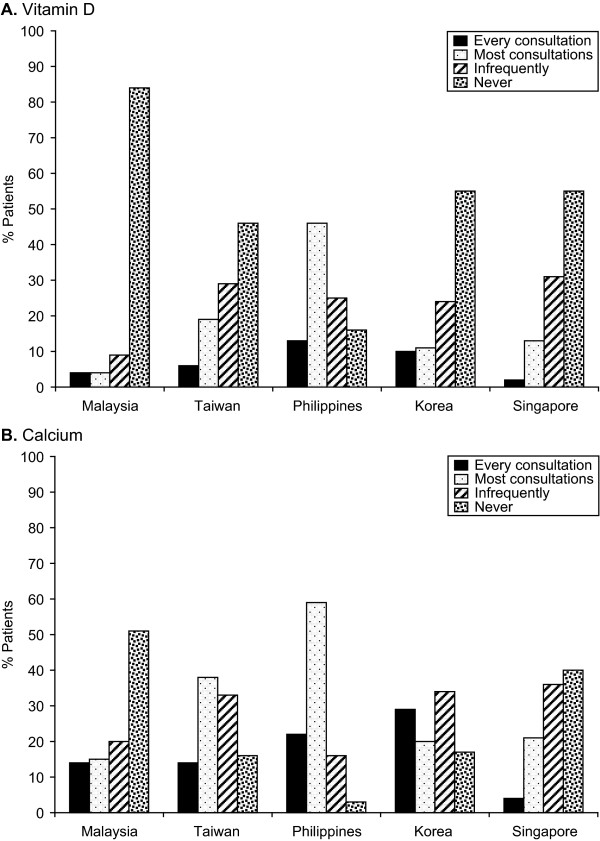
**A: Frequency at which Vitamin D is discussed; Frequency at which Calcium is discussed**.

In Malaysia, only 13% of patients reported taking both calcium and vitamin D supplements either separately (6%) or together in a combination pill (7%) (Figure-3). However, 83% of those patients who take supplements reported taking supplements recommended by their doctor daily, while 3% reported taking supplements most days (Figure 4). Fifty-one percent of physicians estimated that their patients were fully compliant and took their supplements, which included vitamin D, daily, while 27% estimated compliance at most days (Figure 5). 84% and 51% of the patients reported that their physician never discussed the importance of vitamin D and calcium, respectively, in relation to the management of their osteoporosis (Figure 6a and 6b).

#### Taiwan

Thirty percent of patients in Taiwan reported taking both calcium and vitamin D supplements either separately (8%) or together in a combination pill (22%) (Figure 3), and of those, 91% took the supplements on a regular basis (83% every day; 9% most days) (Figure 4). Eighteen percent of physicians estimated that their patients were fully compliant and took their supplements daily, while 16% estimated compliance on most days (Figure 5). The majority of patients in Taiwan (75%) reported that they had had no (46%) or infrequent (29%) discussions with their physicians about vitamin D, and almost 50% of patients reported, no (16%) or infrequent (33%) discussions with their physicians about calcium (Figure 6a and 6b).

#### The Philippines

In the Philippines, 55% of patients reported taking both calcium and vitamin D supplements either separately (45%) or together in a combination pill (10%) (Figure 3), with 98% taking supplements on a regular basis (93% every day; 5% most days) (Figure 4). Eighty-four percent of physicians estimated that their patients were fully compliant and took their supplements daily, while 6% estimated compliance on most days (Figure 5). Less than half of the patients reported that they had had no (16%) or infrequent (25%) discussions with their physicians regarding vitamin D, while 3% reported no and 16% reported infrequent discussions about calcium (Figure 6a and 6b).

#### Korea

In Korea, 23% of patients reported taking both calcium and vitamin D supplements either separately (9%) or together in a combination pill (14%) (Figure 3), with 81% taking supplements on a regular basis (73% every day; 8% on most days) (Figure 4). Physicians reported that only 14% of their patients take their supplements daily and 12% take their supplements most days (Figure 5). The majority of patients in Korea (75%) reported that they had had no (55%) or infrequent (24%) discussions with their physicians about vitamin D and 51% of patients reported, no (17%) or infrequent (34%) discussions with their physicians about calcium (Figure-6a and 6b)

#### Singapore

In Singapore, 69% of patients reported taking both calcium and vitamin D supplements either separately (46%) or together in a combination pill (23%) (Figure 3), with 91% of those doing so on a regular basis (76% every day; 15% on most days) (Figure 4). Physicians reported that 42% of their patients take their supplements daily, while 50% take their supplements on most days (Figure 5). The majority of patients in Singapore (86%) reported that they had had no (55%) or infrequent (31%) discussions with their physicians regarding vitamin D, and 76% of patients reported, no (40%) or infrequent (36%) discussions with their physicians about calcium (Figure 6a and 6b).

## Discussion

Supplementation of dietary calcium and vitamin D plays a critical role in the therapy of osteoporosis [20,53]. Given the essential role of vitamin D in calcium absorption from the gut, any insufficiency in vitamin D negatively impacts calcium metabolism irrespective of intake of sufficient dietary or supplemental calcium [40,45]. Therefore insufficient and sporadic intake of vitamin D can lead to chronic vitamin D deficiency/insufficiency, thereby reducing the effectiveness of osteoporosis treatment [40,45].

The outcome of this study has demonstrated that physicians in Malaysia, Taiwan and Singapore had a poor appreciation of the importance of vitamin D and calcium for bone health, physicians in Korea had a fair appreciation of it and physicians in the Philippines had a heightened appreciation of the importance of both for bone health. In general, there was a good understanding of the importance of vitamin D and calcium among patients from these five countries, although it was evident that patients considered calcium to be more important than vitamin D.

Given the poor understanding among physicians and patients that vitamin D is central to maintenance of bone health; it is not surprising that the percentage of patients who frequently took vitamin D supplements was low. In Malaysia, Taiwan and Korea, less than 30% of patients reported taking vitamin D and calcium.

Although the questionnaire highlighted, with the exception of the Philippines, a poor awareness among physicians of the importance of calcium and, in particular, vitamin D in osteoporosis management, this resulted in poor advice for patients, as at least 50% of patients in Malaysia, Taiwan, Korea, and Singapore reported no or infrequent discussions with their physicians regarding the importance of vitamin D and calcium supplementation.

Of these five countries, the Philippines clearly stood out as the country where both physicians and patients understood the importance of both vitamin D and calcium in the management of osteoporosis. This understanding resulted in a high compliance among patients for supplements to be taken with osteoporosis treatments.

This study provides additional evidence that in Asian countries compliance with treatment guidelines is not optimal among osteoporosis patients that may be at high risk for vitamin D insufficiency/deficiency [58-60]. The prevalence of vitamin D insufficiency is high in South Asian countries [14,16] and although it is highly unlikely that poor advice from physicians regarding vitamin D supplementation is the sole cause, it is possible that this may be a contributory factor.

In accordance with a number of other studies, this study clearly demonstrates that physicians generally misjudge their patients' compliance with their osteoporosis treatment and their readiness to adhere to treatment directions. The largest discrepancies between physician estimates and patient-reported compliance were in Taiwan and Korea, where 83% and 73% of patients who take supplements, respectively, state that they take them every day, whereas only 18% of physicians in Taiwan and 14% in Korea estimated their patients were fully compliant. In Malaysia these figures were 83% of patients vs. 51% of physicians; in Singapore 76% of patients vs. 42% of physicians. The exception was in the Philippines, where 93% of patients and 84% of physicians reported compliance.

It is possible that this effect is because physicians are hesitant to state that their patients take their vitamin D and calcium supplements every day. By combining the responses for compliance most days with compliance every day, then the results are more similar (86% of patients vs. 78% of physicians in Malaysia; 98% vs. 90% in the Philippines; 91% vs. 92% in Singapore); however, even after combining these responses, the difference between patient- and physician-reported compliance in Taiwan and Korea was significant.

Responses to the questionnaire by patients about how the frequently their physicians discussed vitamin D and calcium with them revealed that, in each case, half of the patients reported that fewer discussions occurred than their physicians reported (data not shown). Interestingly, results from the Philippines demonstrated the greatest agreement between patient and physicians of all the countries studied in the region. In addition, patients in the Philippines not only reported discussions with physicians at a frequency similar to that reported by the physicians themselves, the patients also reported the occurrence of discussions about vitamin D and calcium at a higher level than the other countries studied. In all countries studied, patients reported that calcium was discussed more frequently than vitamin D.

It is generally accepted that patients have improved health outcomes when they are better informed and are involved their treatment [61]. The patients' compliance with treatment is improved when the reason for treatment is explained in combination with details of the potential effectiveness of the therapies [61].

The findings from this study advocate that there is a need to enhance patients' and physicians' communication regarding the importance of vitamin D and calcium supplementation in treating osteoporosis. Evidence supporting this conclusion can be observed clearly in the study findings from the Philippines, where there was the best patient-reported communication from physicians regarding vitamin D and calcium. This led to an increased understanding of the importance of vitamin D and calcium in the treatment of osteoporosis and increased patient compliance for both supplementary vitamin D and calcium.

Although a lack of information may lead to a lack of compliance in addition to a lack of understanding, compliance may also be particularly low among elderly osteoporosis patients [62]. It has been reported that two-thirds of aged patients with hip fracture who were recruited into a postsurgical follow-up program had low levels of vitamin D. Even after consultation explaining the necessity of calcium and vitamin D supplementation in osteoporosis therapy, the majority of these patients were still not compliant with the recommended guidelines 3 months after discharge from hospital^55^. A potential reason for this observation may be declining compliance as the number of medications increases, and older patients frequently take several medications to treat a range of comorbidities [61,63,64]. The average age of the patients in our study was 63.4 years.

## Limitations

The degree to which these results are applicable to the entire Asian population is unclear. Although the questionnaire was completed by a relatively large number of patients and physicians, care must be used when applying the results to the entire Asian population, since participating physicians were chosen from among specific specialty databases, instead of randomly selected from the entire physician population in Malaysia, Taiwan, the Philippines, Korea and Singapore. This study utilized patients' self-reporting of their knowledge of, use of and compliance with vitamin D and calcium. It is frequently the case that self-reporting of compliance has a tendency to be elevated. Another possible limitation of this study was the inability to determine baseline beliefs regarding the severity and/or perceived susceptibility related to osteoporosis. Despite these possible limitations, the results are in agreement with other reports[27,46-48] and underscore the importance of improving the management of osteoporosis by increasing patient and physician understanding of the importance of vitamin D and calcium in maintaining bone health.

## Conclusions

The effect of therapies for osteoporosis can be maximized by the addition of supplementary vitamin D and calcium to the diet. It appears that physicians and patients, with the notable exception of the Philippines, lack knowledge of the potential benefits of calcium and vitamin D supplementation, and consequently, compliance with the dosing regimen was observed to be insufficient. Although the insidious nature of this disease, the paucity of immediate perceptible benefit and an inadequate patient motivation may be reasons to explain reduced compliance, this questionnaire underlines the need to improve the way this information is communicated to patients and physicians, to define more effective educational and behavioral involvement, and to design additional studies to identify the underlying reasons for the disparities between the recognized benefits of supplementary calcium and vitamin D and the low compliance with these by osteoporosis patients.

## Competing interests

The questionnaire was sponsored by Merck Sharpe & Dohme (MSD) and conducted by Research International Healthcare and Ogilvy Public Relations Worldwide. The questionnaire was designed by MSD in collaboration with Research International Healthcare and Ogilvy Public Relations Worldwide. The questionnaire was performed, data collected and analyzed by Research International Healthcare and Ogilvy Public Relations Worldwide.

SSS and BBS are employees of Merck & Co., Inc. and potentially have stock or other ownership interests in the company.

## Authors' contributions

SCP interpreted the results wrote sections of the initial draft and provided substantive suggestions for revision on subsequent iterations of the manuscript. BBS interpreted the results, wrote sections of the initial draft and provided substantive suggestions for revision on subsequent iterations of the manuscript. SSS conceived and designed the study, and provided substantive suggestions for revision on subsequent iterations of the manuscript. All authors read and approved the final manuscript.

## Pre-publication history

The pre-publication history for this paper can be accessed here:

http://www.biomedcentral.com/1471-2474/11/248/prepub

## Supplementary Material

Additional file 1 **Osteoporosis patient questionnaire**. This file contains the questionnaire that was used for patients in this study. Click here for file

Additional file 2 **Osteoporosis physician questionnaire**. This file contains the questionnaire that was used for physicians in this study. Click here for file

## References

[B1] Johnell O The socioeconomic burden of fractures: today and in the 21st century Am J Med 1997 103 20S 25S 10.1016/S0002-9343(97)90023-1 9302894

[B2] Johnell O Kanis JA An estimate of the worldwide prevalence and disability associated with osteoporotic fractures Osteoporos Int 2006 17 1726 1733 10.1007/s00198-006-0172-4 16983459

[B3] Limpaphayom KK Taechakraichana N Jaisamrarn U Bunyavejchevin S Chaikittisilpa S Poshyachinda M Prevalence of osteopenia and osteoporosis in Thai women Menopause 2001 8 65 69 10.1097/00042192-200101000-00011 11201518

[B4] Lynn HS Lau EM Au B Leung PC Bone mineral density reference norms for Hong Kong Chinese Osteoporos Int 2005 16 1663 1668 10.1007/s00198-005-1899-z 16027958

[B5] Nguyen HT von Schoultz B Pham DM Nguyen DB Le QH Nguyen DV Peak bone mineral density in Vietnamese women Arch Osteoporos 2009 4 9 15 10.1007/s11657-009-0021-0 20234855 PMC2836743

[B6] Gullberg B Johnell O Kanis JA World-wide projections for hip fracture Osteoporos Int 1997 7 407 413 10.1007/PL00004148 9425497

[B7] Lau EM Lee JK Suriwongpaisal P Saw SM Das DS Khir A The incidence of hip fracture in four Asian countries: the Asian Osteoporosis Study (AOS) Osteoporos Int 2001 12 239 243 10.1007/s001980170135 11315243

[B8] Melton LJ III Gabriel SE Crowson CS Tosteson AN Johnell O Kanis JA Cost-equivalence of different osteoporotic fractures Osteoporos Int 2003 14 383 388 10.1007/s00198-003-1385-4 12730750

[B9] Lau EM Suriwongpaisal P Lee JK Das DS Festin MR Saw SM Risk factors for hip fracture in Asian men and women: the Asian osteoporosis study J Bone Miner Res 2001 16 572 580 10.1359/jbmr.2001.16.3.572 11277276

[B10] Wu XP Liao EY Huang G Dai RC Zhang H A comparison study of the reference curves of bone mineral density at different skeletal sites in native Chinese, Japanese, and American Caucasian women Calcif Tissue Int 2003 73 122 132 10.1007/s00223-002-1069-7 14565593

[B11] Chie WC Yang RS Liu JP Tsai KS High incidence rate of hip fracture in Taiwan: estimated from a nationwide health insurance database Osteoporos Int 2004 15 998 1002 10.1007/s00198-004-1651-0 15156304

[B12] Koh LK An Asian perspective to the problem of osteoporosis Ann Acad Med Singapore 2002 31 26 29 11885491

[B13] Wong MK Arjandas Ching LK Lim SL Lo NN Osteoporotic hip fractures in Singapore--costs and patient's outcome Ann Acad Med Singapore 2002 31 3 7 11885492

[B14] Mithal A Wahl DA Bonjour JP Burckhardt P Dawson-Hughes B Eisman JA Global vitamin D status and determinants of hypovitaminosis D Osteoporos Int 2009 20 1807 1820 10.1007/s00198-009-0954-6 19543765

[B15] Ho-Pham LT Nguyen ND Lai TQ Eisman JA Nguyen TV Vitamin D status and parathyroid hormone in a urban population in Vietnam Osteoporos Int 2010 20414642 10.1007/s00198-010-1207-4

[B16] Rahman SA Chee WS Yassin Z Chan SP Vitamin D status among postmenopausal Malaysian women Asia Pac J Clin Nutr 2004 13 255 260 15331337

[B17] McClung MR Bisphosphonates in osteoporosis: recent clinical experience Expert Opin Pharmacother 2000 1 225 238 10.1517/14656566.1.2.225 11249544

[B18] Huang Z Himes JH McGovern PG Nutrition and subsequent hip fracture risk among a national cohort of white women Am J Epidemiol 1996 144 124 134 8678043 10.1093/oxfordjournals.aje.a008899

[B19] Kemmler W Lauber D Weineck J Hensen J Kalender W Engelke K Benefits of 2 years of intense exercise on bone density, physical fitness, and blood lipids in early postmenopausal osteopenic women: results of the Erlangen Fitness Osteoporosis Prevention Study (EFOPS) Arch Intern Med 2004 164 1084 1091 10.1001/archinte.164.10.1084 15159265

[B20] Boonen S Vanderschueren D Haentjens P Lips P Calcium and vitamin D in the prevention and treatment of osteoporosis - a clinical update J Intern Med 2006 259 539 552 10.1111/j.1365-2796.2006.01655.x 16704554

[B21] Hirota K Hirota T [Nutritional condition affect the potency of pharmacological therapy] Clin Calcium 2005 15 1535 1539 16137955

[B22] Obermayer-Pietsch BM Bonelli CM Walter DE Kuhn RJ Fahrleitner-Pammer A Berghold A Genetic predisposition for adult lactose intolerance and relation to diet, bone density, and bone fractures J Bone Miner Res 2004 19 42 47 10.1359/jbmr.0301207 14753735

[B23] Devine A Prince RL Bell R Nutritional effect of calcium supplementation by skim milk powder or calcium tablets on total nutrient intake in postmenopausal women Am J Clin Nutr 1996 64 731 737 8901793 10.1093/ajcn/64.5.731

[B24] Devine A Dick IM Heal SJ Criddle RA Prince RL A 4-year follow-up study of the effects of calcium supplementation on bone density in elderly postmenopausal women Osteoporos Int 1997 7 23 28 10.1007/BF01623455 9102058

[B25] Recker RR Hinders S Davies KM Heaney RP Stegman MR Lappe JM Correcting calcium nutritional deficiency prevents spine fractures in elderly women J Bone Miner Res 1996 11 1961 1966 10.1002/jbmr.5650111218 8970899

[B26] Reid IR Ames RW Evans MC Gamble GD Sharpe SJ Long-term effects of calcium supplementation on bone loss and fractures in postmenopausal women: a randomized controlled trial Am J Med 1995 98 331 335 10.1016/S0002-9343(99)80310-6 7709944

[B27] Shea B Wells G Cranney A Zytaruk N Robinson V Griffith L Meta-analyses of therapies for postmenopausal osteoporosis. VII. Meta-analysis of calcium supplementation for the prevention of postmenopausal osteoporosis Endocr Rev 2002 23 552 559 10.1210/er.2001-7002 12202470

[B28] Lips P Graafmans WC Ooms ME Bezemer PD Bouter LM Vitamin D supplementation and fracture incidence in elderly persons. A randomized, placebo-controlled clinical trial Ann Intern Med 1996 124 400 406 8554248 10.7326/0003-4819-124-4-199602150-00003

[B29] Lips P Vitamin D deficiency and secondary hyperparathyroidism in the elderly: consequences for bone loss and fractures and therapeutic implications Endocr Rev 2001 22 477 501 10.1210/er.22.4.477 11493580

[B30] Bischoff HA Stahelin HB Dick W Akos R Knecht M Salis C Effects of vitamin D and calcium supplementation on falls: a randomized controlled trial J Bone Miner Res 2003 18 343 351 10.1359/jbmr.2003.18.2.343 12568412

[B31] Chapuy MC Arlot ME Duboeuf F Brun J Crouzet B Arnaud S Vitamin D3 and calcium to prevent hip fractures in the elderly women N Engl J Med 1992 327 1637 1642 10.1056/NEJM199212033272305 1331788

[B32] Chapuy MC Pamphile R Paris E Kempf C Schlichting M Arnaud S Combined calcium and vitamin D3 supplementation in elderly women: confirmation of reversal of secondary hyperparathyroidism and hip fracture risk: the Decalyos II study Osteoporos Int 2002 13 257 264 10.1007/s001980200023 11991447

[B33] Dawson-Hughes B Dallal GE Krall EA Harris S Sokoll LJ Falconer G Effect of vitamin D supplementation on wintertime and overall bone loss in healthy postmenopausal women Ann Intern Med 1991 115 505 512 1883119 10.7326/0003-4819-115-7-505

[B34] Rossini M Bianchi G Di Munno O Giannini S Minisola S Sinigaglia L Determinants of adherence to osteoporosis treatment in clinical practice Osteoporos Int 2006 17 914 921 10.1007/s00198-006-0073-6 16538553

[B35] FAO/WHO expert consultation on human vitamin and mineral requirements ftp://ftp.fao.org/es/esn/nutrition/Vitrni/vitrni.html

[B36] Standing Committee on the Scientific Evaluation of Dietary Reference Intakes Food and Nutrition Board Institute of Medicine Dietary Reference Intakes for Calcium Phosphorus Magnesium, Vitamin D, and Fluoride 1997 Washington, D.C.: National Academy Press

[B37] Francis RM Anderson FH Patel S Sahota O van Staa TP Calcium and vitamin D in the prevention of osteoporotic fractures QJM 2006 99 355 363 10.1093/qjmed/hcl031 16537574

[B38] Uenishi K [Prevention of osteoporosis by foods and dietary supplements. Prevention of osteoporosis by milk and dairy products] Clin Calcium 2006 16 17012811

[B39] Holick MF Siris ES Binkley N Beard MK Khan A Katzer JT Prevalence of Vitamin D inadequacy among postmenopausal North American women receiving osteoporosis therapy J Clin Endocrinol Metab 2005 90 3215 3224 10.1210/jc.2004-2364 15797954

[B40] Heaney RP Dowell MS Hale CA Bendich A Calcium absorption varies within the reference range for serum 25-hydroxyvitamin D J Am Coll Nutr 2003 22 142 146 12672710 10.1080/07315724.2003.10719287

[B41] World Health Organization Collaborating Centre; International Osteoporosis Foundation (IOF) Committee of Scientific Advisors Invest in your bones. Osteoporosis in the workplace: the social, economic and human costs of osteoporosis on employee, employers and governments 2002 Liege (Belgium)

[B42] European Commission Health and Consumer Protection Directorate-General: Scientific Committee on Food. Opinion of the Scientific Committee on Food on the tolerable upper intake level of vitamin D 2003 http://ec.europa.eu/food/fs/sc/scf/out196_en.pdf

[B43] Lips P Duong T Oleksik A Black D Cummings S Cox D A global study of vitamin D status and parathyroid function in postmenopausal women with osteoporosis: baseline data from the multiple outcomes of raloxifene evaluation clinical trial J Clin Endocrinol Metab 2001 86 1212 1221 10.1210/jc.86.3.1212 11238511

[B44] Holick MF Vitamin D: A millenium perspective J Cell Biochem 2003 88 296 307 10.1002/jcb.10338 12520530

[B45] Holick MF High prevalence of vitamin D inadequacy and implications for health Mayo Clin Proc 2006 81 353 373 10.4065/81.3.353 16529140

[B46] Weaver CM Fleet JC Vitamin D requirements: current and future Am J Clin Nutr 2004 80 1735S 1739S 15585797 10.1093/ajcn/80.6.1735S

[B47] Lips P Hosking D Lippuner K Norquist JM Wehren L Maalouf G The prevalence of vitamin D inadequacy amongst women with osteoporosis: an international epidemiological investigation J Intern Med 2006 260 245 254 10.1111/j.1365-2796.2006.01685.x 16918822

[B48] Lips P Binkley N Pfeifer M Recker R Samanta S Cohn DA Once-weekly dose of 8400 IU vitamin D3 compared with placebo: effects on neuromuscular function and tolerability in older adults with vitamin D insufficiency Am J Clin Nutr 2010 91 985 991 10.3945/ajcn.2009.28113 20130093

[B49] Gallacher SJ McQuillian C Harkness M Finlay F Gallagher AP Dixon T Prevalence of vitamin D inadequacy in Scottish adults with non-vertebral fragility fractures Curr Med Res Opin 2005 21 1355 1361 10.1185/030079905X59148 16197653

[B50] Gaugris S Heaney RP Boonen S Kurth H Bentkover JD Sen SS Vitamin D inadequacy among post-menopausal women: a systematic review QJM 2005 98 667 676 10.1093/qjmed/hci096 16006498

[B51] Moniz C Dew T Dixon T Prevalence of vitamin D inadequacy in osteoporotic hip fracture patients in London Curr Med Res Opin 2005 21 1891 1894 10.1185/030079905X75023 16368037

[B52] Grant WB Holick MF Benefits and requirements of vitamin D for optimal health: a review Altern Med Rev 2005 10 94 111 15989379

[B53] Prasad N Sunderamoorthy D Martin J Murray JM Secondary prevention of fragility fractures: are we following the guidelines? Closing the audit loop Ann R Coll Surg Engl 2006 88 470 474 10.1308/003588406X116891 17002853 PMC1964691

[B54] Adami S Isaia G Luisetto G Minisola S Sinigaglia L Gentilella R Fracture incidence and characterization in patients on osteoporosis treatment: the ICARO study J Bone Miner Res 2006 21 1565 1570 10.1359/jbmr.060715 16995811

[B55] KRC Research International Osteoporosis Foundation Survey 2007 http://www.iofbonehealth.org/publications.html

[B56] IPSOS Health European survey of physicians and women with osteoporosis Sponsored by Roche/GSK 2005 http://www.iofbonehealth.org/download/osteofound/filemanager/publications/pdf/adherence_gap_report.pdf

[B57] Resch H Walliser J Phillips S Wehren LE Sen SS Physician and patient perceptions on the use of vitamin D and calcium in osteoporosis treatment: a European and Latin American perspective Curr Med Res Opin 2007 23 1227 1237 10.1185/030079907X187964 17559732

[B58] Kung AW Lee KK Knowledge of vitamin D and perceptions and attitudes toward sunlight among Chinese middle-aged and elderly women: a population survey in Hong Kong BMC Public Health 2006 6 226 10.1186/1471-2458-6-226 16956420 PMC1584409

[B59] Lim SK Kung AW Sompongse S Soontrapa S Tsai KS Vitamin D inadequacy in postmenopausal women in Eastern Asia Curr Med Res Opin 2008 24 99 106 10.1185/030079908X253429 18028585

[B60] Wat WZ Leung JY Tam S Kung AW Prevalence and impact of vitamin D insufficiency in southern Chinese adults Ann Nutr Metab 2007 51 59 64 10.1159/000100822 17356256

[B61] Osterberg L Blaschke T Adherence to medication N Engl J Med 2005 353 487 497 10.1056/NEJMra050100 16079372

[B62] Segal E Zinnman H Raz B Tamir A Ish-Shalom S Adherence to vitamin D supplementation in elderly patients after hip fracture J Am Geriatr Soc 2004 52 474 475 10.1111/j.1532-5415.2004.52125_8.x 14962175

[B63] Balkrishnan R Predictors of medication adherence in the elderly Clin Ther 1998 20 764 771 10.1016/S0149-2918(98)80139-2 9737835

[B64] Vlasnik JJ Aliotta SL DeLor B Medication adherence: factors influencing compliance with prescribed medication plans Case Manager 2005 16 47 51 10.1016/j.casemgr.2005.01.009 15818344

