# Supplementary material for: An Asian viewpoint on the use of vitamin D and calcium in osteoporosis treatment: Physician and patient attitudes and beliefs
Source: BMC Musculoskelet Disord. 2010 Oct 26;11:248. doi: 10.1186/1471-2474-11-248 (PMC2987973; doi:10.1186/1471-2474-11-248)
Supplement: Additional file 1 — Osteoporosis patient questionnaire. This file contains the questionnaire that was used for patients in this study. [file 1471-2474-11-248-S1.DOC]

| 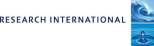 | **Project Osteoporosis**  **RI 40361111**  **Version 2.0**  April 2005 | | FOR OFFICE USE ONLY  SERIAL No. (201-204)  (205-206): 01 |
| --- | --- | --- | --- |
|  |  |  |  |
|  | Approved by COG |  |  |
|  | Approved by DP |  |  |
|  | Approved by Exec |  |  |

| Unique Identifier  Interview Date | | | | | |
| --- | --- | --- | --- | --- | --- |
| (207) | | | | | |
| Country | Hong Kong | | | 1 |  |
|  | Korea | | | 2 |  |
|  | Malaysia | | | 3 |  |
|  | Singapore | | | 4 |  |
|  | Taiwan | | | 5 |  |
|  | Philippines | | | 6 |  |
| **Physician Number** | | | | |  |
| Time Interview Began Ended ***(Enter interview length at the END of the q’naire)*** | | | | | |
| I declare that this interview has been carried out strictly in accordance with your specification and has been conducted within the MRS Code of Conduct with a person unknown to me. | | Interviewers signature. | Checked by supervisor | | |

|  | INSERT 4 DIGIT PHYSICIAN SERIAL NO  (208-211) |  | (212-213) |
| --- | --- | --- | --- |
| Physician serial |  | Patient number | 01 |
|  |  |  | 02 |
|  |  |  | 03 |
|  |  |  | 04 |
|  |  |  | 05 |
|  |  |  | 06 |
|  |  |  | 07 |
|  |  |  | 08 |

This interview will be looking at the way you deal with Osteoporosis, also known as weak, thinning or brittle bones. We will be asking you questions related to your experiences specifically.

We would like to remind you that there are a number of patients from your doctor’s office, as well as patients from other doctors, who will be answering this questionnaire and you have all been selected at random. Your answers will not be shared with your physician or anyone else and will be kept confidential so please be honest with your responses.

Q1 What do you do for your Osteoporosis?

*[If necessary, prompt the respondent with: “For instance, do you take medications or have you changed your lifestyle?]*

OPEN RESPONSE (write in)

| (214) | (215) | (216) | (217) | (218) | (219) | (220) | (221) | (222) | (223) | (224) | (225) | (226) | (227) | (228) | (229) | (230) | (231) | (232) | (233) |
| --- | --- | --- | --- | --- | --- | --- | --- | --- | --- | --- | --- | --- | --- | --- | --- | --- | --- | --- | --- |
|  |  |  |  |  |  |  |  |  |  |  |  |  |  |  |  |  |  |  |  |

**RECORD AS DOUBLE DIGIT CODES USING LEADING ZEROES**

Q2 Are you taking supplements for vitamin D or Calcium to treat your osteoporosis?

**Code 1 response only**

(234)

Yes, for **vitamin D supplements** 1

Yes, for **Calcium supplements** 2

Yes, for both **Calcium and vitamin D supplements** 3

Yes, for **Calcium and vitamin D in a combination** pill 4

No 5

**If Q2 is coded 1-4, ask Q3. If Q2 is coded 5, skip to Q5.**

**INSERT ONLY BOLDED PORTION OF Q2**

Q3 On average, how often do you take your [INSERT ANSWER FROM Q2] as recommended by your doctor?

**Code 1 response only**

(235)

Every day (once or twice-a-day)…………………………….1

Most days 2

About half the time 3

Erratically/infrequently 4

Never 5

My doctor does not recommend supplements 6

**If Q3 is coded 2-5, ask Q4. If Q3 is coded 1, skip to Q5.**

**INSERT ONLY BOLDED PORTION OF Q2**

Q4 What is the main reason you do not take your [INSERT ANSWER FROM Q2] as recommended?

**Code one response only**

*Rotate and tick start*

(236-237)

I can get enough calcium through diet 01

I can get enough vitamin D through diet 02

I tend to forget 03

I run out of supplements 04

I take many medications at different times and forget 05

It causes constipation 06

Other (Please specify below) 07

ASK ALL

Q5 Please respond to the following statements as True or False:

*Rotate and tick start*

True False

Calcium is a vital part of my Osteoporosis treatment 1 2 (238)

Vitamin D is a vital part of my Osteoporosis treatment 1 2 (239)

Calcium helps the body to absorb Vitamin D 1 2 (240)

Vitamin D helps the body to absorb Calcium 1 2 (241)

I can get enough vitamin D through a healthy diet 1 2 (242)

I can get enough calcium through a healthy diet 1 2 (243)

Q6 Which of the following do you think are good sources of vitamin D?

**Please respond yes or no to each option**

*Rotate and tick start*

Yes No

Milk 1 2 (244)

Oily fish such as sardines 1 2 (245)

Yoghurt and cheese 1 2 (246)

Citrus fruits such as grapefruit and oranges 1 2 (247)

Sunlight 1 2 (248)

Green leafy vegetables 1 2 (249)

Vitamin supplements containing vitamin D 1 2 (250)

Red meat 1 2 (251)

Q7 Thinking of your Osteoporosis, how would you rate the following in terms of building strong and healthy bones?

Please give your answer on a scale of 1 to 10 where 1 is not at all important and 10 is extremely important

*Rotate and tick start*

Calcium [____] (252-253)

Vitamin D [____] (254-255)

Diet [____] (256-257)

Exercise [____] (258-259)

Q8a When speaking to your doctor about your osteoporosis, how often do you discuss the importance of Calcium?

**Code 1 response only**

(260)

Every consultation 1

Most consultations 2

Infrequently 3

Never 4

Q8b When speaking to your doctor about your osteoporosis, how often do you discuss the importance of Vitamin D?

**Code 1 response only**

(261)

Every consultation 1

Most consultations 2

Infrequently 3

Never 4

**READ OUT:** The next set of questions I will ask you will focus on Calcium

**If Q8a is coded 1-3, ask Q9. If Q8a is coded 4, skip to Q11.**

Q9 Thinking about the consultations when you discuss the importance of Calcium with your physician, how often do you initiate these conversations?

**Code 1 response only**

(262)

Every time 1

Most of the time 2

Never 3

Q10 Thinking about the consultations when you discuss the importance of Calcium with your physician, how often does your physician initiate these conversations?

**Code 1 response only**

(263)

Every time 1

Most of the time 2

Never 3

Q11 How would you rate the time spent talking about Calcium with your doctor?

**Code 1 response only**

(264)

Too much time is spent talking about calcium 1

An appropriate amount of time is spent talking about calcium 2

Not enough time is spent talking about calcium 3

We do not talk about calcium in my consultations 4

Q12 How well do you feel your doctor explains the role of Calcium in the treatment of osteoporosis?

**Code 1 response only**

(265)

Extremely well – I understand all the information given 1

Moderately well – I understand some of the information given 2

Not well enough – I do not understand the information given 3

He/she has never explained the role of Calcium 4

Q13 When your doctor discusses Calcium with you, what type of information is most useful?

**Code 1 response only**

(266)

Verbal advice during consultation 1

Patient information leaflets 2

Dietary information publications 3

Relevant websites 4

Other (please specify) 5

My doctor does not discuss Calcium with me 6

**READ OUT:** The next set of questions I will ask you will focus on Vitamin D

**If Q8b is coded 1-3, ask Q14. If Q8b is coded 4, skip to Q16.**

Q14 Thinking about the consultations when you discuss the importance of Vitamin D with your physician, how often do you initiate these conversations?

**Code 1 response only**

(267)

Every time 1

Most of the time 2

Never 3

Q15 Thinking about the consultations when you discuss the importance of Vitamin D with your physician, how often does your physician initiate these conversations?

**Code 1 response only**

(268)

Every time 1

Most of the time 2

Never 3

Q16 How would you rate the time spent talking about Vitamin D with your doctor?

**Code 1 response only**

(269)

Too much time is spent talking about Vitamin D 1

An appropriate amount of time is spent talking about Vitamin D 2

Not enough time is spent talking about Vitamin D 3

We do not talk about Vitamin D in my consultations 4

Q17 How well do you feel your doctor explains the role of Vitamin D in the treatment of osteoporosis?

**Code 1 response only**

(270)

Extremely well – I understand all the information given 1

Moderately well – I understand some of the information given 2

Not well enough – I do not understand the information given 3

He/she has never explained the role of Vitamin D 4

Q18 When your doctor discusses Vitamin D with you, what type of information is most useful?

**Code 1 response only**

(271)

Verbal advice during consultation 1

Patient information leaflets 2

Dietary information publications 3

Relevant websites 4

Other (please specify) 5

My doctor does not discuss Vitamin D with me 6

Ask Q19 if respondent answered 1, 2 or 3 to Q8a.

Q19 What recommendations does your doctor make with regard to Calcium?

*[If necessary, prompt the respondent with: “For instance, does your doctor recommend a particular product/dosage/diet? Please ensure that you probe about the dosage”]*

OPEN RESPONSE (write in)

| (272) | (273) | (274) | (275) | (276) | (277) | (278) | (279) | (307) | (308) | (309) | (310) | (311) | (312) | (313) | (314) | (315) | (316) | (317) | (318) |
| --- | --- | --- | --- | --- | --- | --- | --- | --- | --- | --- | --- | --- | --- | --- | --- | --- | --- | --- | --- |
|  |  |  |  |  |  |  |  |  |  |  |  |  |  |  |  |  |  |  |  |

**RECORD AS DOUBLE DIGIT CODES USING LEADING ZEROES**

Ask Q20 if respondent answered 1, 2 or 3 to Q8b.

Q20 What recommendations does your doctor make with regard to Vitamin D?

*[If necessary, prompt the respondent with: “For instance, does your doctor recommend a particular product/dosage/diet? Please ensure that you probe about the dosage”]*

OPEN RESPONSE (write in)

| (319) | (320) | (321) | (322) | (323) | (324) | (325) | (326) | (327) | (328) | (329) | (330) | (331) | (332) | (333) | (334) | (335) | (336) | (337) | (338) |
| --- | --- | --- | --- | --- | --- | --- | --- | --- | --- | --- | --- | --- | --- | --- | --- | --- | --- | --- | --- |
|  |  |  |  |  |  |  |  |  |  |  |  |  |  |  |  |  |  |  |  |

**RECORD AS DOUBLE DIGIT CODES USING LEADING ZEROES**

ASK ALL

Q21 Have you seen a recent report in the media about the role of Calcium & Vitamin D and fractures in the treatment of Osteoporosis?

**Code 1 response only**

(339)

Yes 1

No 2

Ask Q22 if respondent answered “Yes” (1) to Q21.

Q22 How has this affected your perception of the importance of Calcium and Vitamin D?

OPEN RESPONSE (write in)

| (340) | (341) | (342) | (343) | (344) | (345) | (346) | (347) | (348) | (349) | (350) | (351) | (352) | (353) | (354) | (355) | (356) | (357) | (358) | (359) |
| --- | --- | --- | --- | --- | --- | --- | --- | --- | --- | --- | --- | --- | --- | --- | --- | --- | --- | --- | --- |
|  |  |  |  |  |  |  |  |  |  |  |  |  |  |  |  |  |  |  |  |

**RECORD AS DOUBLE DIGIT CODES USING LEADING ZEROES**

IMPORTANT: Please enter interview length below.

|  |  |  | (360-361) |
| --- | --- | --- | --- |
