# Supplementary material for: An Asian viewpoint on the use of vitamin D and calcium in osteoporosis treatment: Physician and patient attitudes and beliefs
Source: BMC Musculoskelet Disord. 2010 Oct 26;11:248. doi: 10.1186/1471-2474-11-248 (PMC2987973; doi:10.1186/1471-2474-11-248)
Supplement: Additional file 2 — Osteoporosis physician questionnaire. This file contains the questionnaire that was used for physicians in this study. [file 1471-2474-11-248-S2.DOC]

| 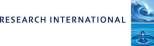 | **Project Osteoporosis**  **RI 40361111**  **Version 1.0**  April 2005 | | FOR OFFICE USE ONLY  SERIAL No. (201-204)  (205-206): 02 |
| --- | --- | --- | --- |
|  |  |  |  |
|  | Approved by COG |  |  |
|  | Approved by DP |  |  |
|  | Approved by Exec |  |  |

| Unique Identifier  Interview Date | | | | | |
| --- | --- | --- | --- | --- | --- |
| (207) | | | | | |
| Country | Hong Kong | | | 1 |  |
|  | Korea | | | 2 |  |
|  | Malaysia | | | 3 |  |
|  | Singapore | | | 4 |  |
|  | Taiwan | | | 5 |  |
|  | Philippines | | | 6 |  |
| **Physician Number** | | | | |  |
| Time Interview Began Ended ***(Enter interview length at the END of the q’naire)*** | | | | | |
| I declare that this interview has been carried out strictly in accordance with your specification and has been conducted within the MRS Code of Conduct with a person unknown to me. | | Interviewers signature. | Checked by supervisor | | |

This interview looks at your current treatment of Osteoporosis with particular reference to the use of supplements including Vitamin D and Calcium.

Q1 Aside from prescription drugs, how important do you rate the following lifestyle changes or supplements for bone health and Osteoporosis?

Please give your answer on a scale of 1 to 10 where 1 is not at all important and 10 is extremely important

*Rotate and tick start*

Calcium [____] (208-209)

Vitamin D [____] (210-211)

Diet [____] (212-213)

Exercise [____] (214-215)

Q2 Aside from prescription drugs, how much emphasis do you place on the following, during an Osteoporosis consultation?

Please give your answer on a scale of 1 to 10 where 1 is not at all important and 10 is extremely important

*Rotate and tick start*

Calcium [____] (216-217)

Vitamin D [____] (218-219)

Diet [____] (220-221)

Exercise [____] (222-223)

Q3a In consultations with your Osteoporosis patients, how often do you discuss the importance of the Calcium in the management of their disease?

**Code 1 response only**

(224)

Every consultation 1

Most consultations 2

Infrequently 3

Never 4

Q3b In consultations with your Osteoporosis patients, how often do you discuss the importance of the Vitamin D in the management of their disease?

**Code 1 response only**

(225)

Every consultation 1

Most consultations 2

Infrequently 3

Never 4

**PLEASE READ OUT:** For the next 3 questions I would like to focus on Calcium.

Q4 When you are in consultation with your Osteoporosis patients, how often do you initiate a discussion about the importance of Calcium in the management of the disease?

**Code 1 response only**

(226)

Every consultation 1

Most of the time 2

Never 3

Q5 When you are in consultation with your Osteoporosis patients, how often do your patients ask about the importance of Calcium in the management of the disease?

**Code 1 response only**

(227)

Every consultation 1

Most of the time 2

Never 3

Q6 If you discuss Calcium when you are in consultation with your Osteoporosis patients, what type of information do you provide to them about Calcium?
**Code all mentioned**

Verbal advice during consultation 1

Patient information leaflets 2

Dietary information publications 3

Direct them to a relevant website 4

Other (Please specify) 5

Other (write in)

| (228) | (229) | (230) | (231) | (232) | (233) | (234) | (235) | (236) | (237) | (238) | (239) | (240) | (241) | (242) | (243) | (244) | (245) | (246) | (247) |
| --- | --- | --- | --- | --- | --- | --- | --- | --- | --- | --- | --- | --- | --- | --- | --- | --- | --- | --- | --- |
|  |  |  |  |  |  |  |  |  |  |  |  |  |  |  |  |  |  |  |  |

**RECORD AS DOUBLE DIGIT CODES USING LEADING ZEROES**

**PLEASE READ OUT:** For the next 3 questions I would like to focus on Vitamin D.

Q7 When you are in consultation with your Osteoporosis patients, how often do you initiate a discussion about the importance of Vitamin D in the management of the disease?

**Code 1 response only**

(248)

Every consultation 1

Most of the time 2

Never 3

Q8 When you are in consultation with your Osteoporosis patients, how often do your patients ask about the importance of Vitamin D in the management of the disease?

**Code 1 response only**

(249)

Every consultation 1

Most of the time 2

Never 3

Q9 If you discuss Calcium when you are in consultation with your Osteoporosis patients, what type of information do you provide to them about Vitamin D?

**Code all mentioned**

*Rotate and tick start*

Verbal advice during consultation 1

Patient information leaflets 2

Dietary information publications 3

Direct them to a relevant website 4

Other (Please specify) 5

Other (write in)

| (250) | (251) | (252) | (253) | (254) | (255) | (256) | (257) | (258) | (259) | (260) | (261) | (262) | (263) | (264) | (265) | (266) | (267) | (268) | (270) |
| --- | --- | --- | --- | --- | --- | --- | --- | --- | --- | --- | --- | --- | --- | --- | --- | --- | --- | --- | --- |
|  |  |  |  |  |  |  |  |  |  |  |  |  |  |  |  |  |  |  |  |

**RECORD AS DOUBLE DIGIT CODES USING LEADING ZEROES**

Q10 Do you believe your osteoporosis patients understand the relationship between Vitamin D and Calcium absorption?

**Code 1 response only**

(271)

Yes, fully 1

They understand enough to manage their condition 2

They have very little understanding 3

Understanding is not essential to successful treatment 4

Not sure/don’t know 5

Q11 What do you recommend to your osteoporosis patients in regards to **Calcium**?

Please be as specific and detailed as possible.

*Interviewer: If necessary, prompt the respondent with: “For instance, do you recommend a particular product/dosage/diet?” Please ensure you prompt about dosage.*

OPEN RESPONSE (write in)

| (272) | (273) | (274) | (275) | (276) | (277) | (278) | (279) | (307) | (308) | (309) | (310) | (311) | (312) | (313) | (314) | (315) | (316) | (317) | (318) |
| --- | --- | --- | --- | --- | --- | --- | --- | --- | --- | --- | --- | --- | --- | --- | --- | --- | --- | --- | --- |
|  |  |  |  |  |  |  |  |  |  |  |  |  |  |  |  |  |  |  |  |

**RECORD AS DOUBLE DIGIT CODES USING LEADING ZEROES**

Q12 What do you recommend to your osteoporosis patients in regards to **Vitamin D**?

Please be as specific and detailed as possible.

*If necessary, prompt the respondent with: “For instance, do you recommend a particular product/dosage/diet?” Please ensure you prompt about dosage.*

OPEN RESPONSE (write in)

| (319) | (320) | (321) | (322) | (323) | (324) | (325) | (326) | (327) | (328) | (329) | (330) | (331) | (332) | (333) | (334) | (335) | (336) | (337) | (338) |
| --- | --- | --- | --- | --- | --- | --- | --- | --- | --- | --- | --- | --- | --- | --- | --- | --- | --- | --- | --- |
|  |  |  |  |  |  |  |  |  |  |  |  |  |  |  |  |  |  |  |  |

**RECORD AS DOUBLE DIGIT CODES USING LEADING ZEROES**

Q13 Considering your average patient, how often do you think they take their vitamin D (or vitamin D plus calcium) supplement?

**Code 1 response only**

(339)

Every day (once or twice-a-day) 1

Most days 2

About half the time 3

Erratically/infrequently 4

Never 5

Q14 Considering your individual patients, how many consistently and accurately follow your recommendations for vitamin D intake in your view?

**Code 1 response only**

(340)

100% 1

80-99% 2

60-79% 3

40-59% 4

20-39% 5

Less than 20% 6

Q15 How many of your patients do you feel are consistently taking at least 400 IU of Vitamin D daily?

Code 1 response only

(341)

100% 1

80-99% 2

60-79% 3

40-59% 4

20-39% 5

Less than 20% 6

Q16 Of those reasons listed below, what do you think is the biggest barrier to patient compliance in regards to taking vitamin D as prescribed?

Code 1 response only

(342-343)

*Rotate and tick start*

Lack of understanding of the importance of vitamin D

in Osteoporosis 01

Misperceptions about the levels obtainable through diet /

exposure to sunlight 02

Forgetting to take each dose as required 03

Running out of supplements/ Failing to fulfill prescription 04

Patients taking numerous medication at different times

leads to non-compliance 05

Vitamin D is in a combination tablet with calcium, which

causes gastro-intestinal upset 06

Don’t know (DO NOT READ) 07

I don’t feel there are any barriers (DO NOT READ) 08

Other (specify) (DO NOT READ) 98

IMPORTANT: Please enter interview length below. (This has moved from the front page)

|  |  |  | (344-345) |
| --- | --- | --- | --- |
